# Adverse Events of Cabozantinib as a Potential Prognostic Factor in Metastatic Renal Cell Carcinoma Patients: Real-World Experience in a Single-Center Retrospective Study

**DOI:** 10.3390/biomedicines12020413

**Published:** 2024-02-09

**Authors:** Piotr Domański, Mateusz Piętak, Barbara Kruczyk, Jadwiga Jarosińska, Anna Mydlak, Tomasz Demkow, Marta Darewicz, Bożena Sikora-Kupis, Paulina Dumnicka, Wojciech Kamzol, Jakub Kucharz

**Affiliations:** 1 Department of Experimental Immunotherapy, Maria Sklodowska-Curie National Research Institute of Oncology, Roentgena 5, 02-781 Warsaw, Poland; 2 Department of Genitourinary Oncology, Maria Sklodowska-Curie National Research Institute of Oncology, Roentgena 5, 02-781 Warsaw, Poland; 3 Department of Head and Neck Oncology, Maria Sklodowska-Curie National Research Institute of Oncology, Roentgena 5, 02-781 Warsaw, Poland; 4 Department of Medical Diagnostics, Jagiellonian University Medical College, 31-008 Cracow, Poland; 5 Department of Radiotherapy, Maria Sklodowska-Curie National Research Institute of Oncology, Roentgena 5, 02-781 Warsaw, Poland

**Keywords:** tyrosine kinase inhibitors (TKI), cabozantinib, metastatic renal cell carcinoma (mRCC), adverse events, predictive factor

## Abstract

Cabozantinib, an oral inhibitor targeting MET, AXL, and VEGF receptors, has become a key component of a sequential treatment strategy for clear cell renal cell carcinoma (ccRCC). The purpose of this work is to show that effective management of adverse events (AEs) during cabozantinib treatment and achieving a balance between AEs and treatment efficacy is crucial to achieving therapeutic goals. In this retrospective study, involving seventy-one metastatic RCC (mRCC) patients receiving second or subsequent lines of cabozantinib at the Department of Genitourinary Oncology, Maria Sklodowska-Curie National Research Institute of Oncology, we explored the impact of AEs on overall survival (OS) and progression-free survival (PFS). AEs were observed in 92% of patients. Hypothyroidism during treatment was significantly associated with prolonged OS and PFS (HR: 0.31; *p* < 0.001 and HR: 0.34; *p* < 0.001, respectively). The occurrence of hand–foot syndrome (HFS) was also linked to improved OS (HR: 0.46; *p* = 0.021). Patients experiencing multiple AEs demonstrated superior OS and PFS compared to those with one or no AEs (HR: 0.36; *p* < 0.001 and HR: 0.30; *p* < 0.001, respectively). Hypothyroidism and HFS serve as valuable predictive factors during cabozantinib treatment in ccRCC patients, indicating a more favorable prognosis.

## 1. Introduction

Renal cell carcinoma (RCC) is the most commonly diagnosed kidney cancers and accounts for 90% of all kidney malignancies [1]. Clear cell carcinoma (ccRCC) is a histological subtype responsible for about 70% of the diagnoses, and it usually develops on a background of different sporadic mutations, including VHL mutation [1]. The VHL mutation results in a loss of control of hypoxia-inducible factor (HIF) expression [1]. Under those circumstances, HIF proteins (HIF-1α, HIF-2α, and HIF-3α) accumulate and, through various molecular pathways, lead to the promotion of angiogenesis and cell proliferation, which play an essential role in carcinogenesis [2,3]. Therefore, it is considered an essential metabolic checkpoint in the development of renal tumors [2].

Understanding these pathways enabled the implementation of tyrosine kinase inhibitors (TKIs) in the systemic treatment of RCC [1]. Cabozantinib is an oral VEGF receptor (VEGFR) inhibitor that also inhibits other receptors and kinases such as RET, KIT, MET, AXL, and ROS1. TKIs targeting VEGFR cause an antiangiogenic effect [4] which limits the growth potential of tumors [1]. Moreover, as VEGF has some immunosuppressive properties, inhibition of VEGFR is responsible for the immunomodulatory properties of cabozantinib, such as decreasing the number of myeloid-derived suppressor cells (MDSC) and regulatory T-cells [5,6,7]. Furthermore, cabozantinib also affects the tumor’s physiology and leads to mutated cell apoptosis, disrupted vascularization, and increased hypoxia within the tumor. Cabozantinib affects not only the tumor microenvironment but also directly acts on the tumor cells, making them more susceptible to immune-mediated killing [8]. These features are the reason for the occurrence of a synergistic effect with immune oncology (IO), which has been already described in various studies [9].

Following the publication of the CABOSUN study, cabozantinib emerged as a viable option for patients falling within the intermediate- and poor-risk categories, as defined by the International Metastatic RCC Database Consortium (IMDC) [10] and, subsequently, the METEOR study [4]. The most recent investigations have demonstrated cabozantinib’s antitumor activity, even in cases where prior therapies involved immune checkpoint inhibitors (IO), combination IO regimens (IOIO), or IO in conjunction with vascular endothelial growth factor inhibitors (VEGFi)-IOVE [11,12,13,14]. The latest clinical guidelines issued by both the European Society for Medical Oncology (ESMO) [15] and the American Society of Clinical Oncology (ASCO) [16] recommend the utilization of combination therapy IO and VEGFi, such as the pairing of nivolumab and cabozantinib. Moreover, cabozantinib is the preferred initial treatment for patients with advanced papillary RCC when additional molecular testing is not deemed necessary [15].

In the treatment of ccRCC with tyrosine kinase inhibitors, AEs have been identified as valuable predictive factors. Previous research has substantiated that AEs such as hand–foot syndrome (HFS), hypothyroidism, or diarrhea are associated with improved overall survival (OS) and progression-free survival (PFS) [17]. Therefore, effectively managing AEs to maintain them at acceptable levels is crucial to ensure the therapeutic effectiveness of this approach. Achieving the right balance between therapeutic efficacy and management of adverse events requires reasonable dose modifications. This becomes particularly significant in the geriatric population. One must bear in mind that cabozantinib is often used as a second-line drug, which often means that there are more geriatric patients in this group than in the group receiving first-line drugs. In those patients, the reduced physiological reserves demand more frequent and delicate adjustments in dosing. The decline in functional reserves and muscle mass is a persistent and inevitable consequence of the ageing process. Furthermore, it affects drug metabolism in the liver and kidney excretion [18,19]. These factors collectively contribute to diminished treatment tolerance, compromised treatment response, and increased susceptibility to treatment-related toxicities.

The early detection of cancer and the implementation of effective radical treatments can delay the need for systemic therapies; therefore, when analyzing the details of cabozantinib treatment, it is imperative to consider all of these aforementioned factors.

## 2. Materials and Methods

### 2.1. Patients Collection

This retrospective analysis included seventy-one patients with biopsy-proven metastatic renal clear cell carcinoma (mRCC) undergoing cabozantinib treatment as a second-line, or further, treatment at the Department of Genitourinary Oncology of the Maria Skłodowska-Curie National Research Institute of Oncology in Warsaw. The database contained the data of patients with mRCC treated at the department between 30 January 2017 and 23 June 2021. This study was performed in line with the principles of the Declaration of Helsinki. Permission to conduct this study was granted by the Maria Sklodowska-Curie National Research Institute of Oncology Bioethics Committee (permission number 38/2018).

### 2.2. Data Collection

The database contained detailed information on age, gender, clinicopathological factors, laboratory results, comorbidities, adverse events, sites of metastases, ECOG performance score, International Metastatic RCC Database Consortium (IMDC), Memorial Sloan-Kettering Cancer Center (MSKCC) risk scores [10], and outcome data associated with individual patients. Characteristics of the studied group at the time of the start of the study are shown in Table 1. Clinical data were extracted from medical records and mortality data were obtained from the Polish national database. This study included patients ranging in age between 42 and 80 years who were treated with cabozantinib as second-line, or further, treatment. The previous lines of treatment are depicted in Table 2. The collected data comprised the date of treatment initiation, type of administered drug, drug dose, date of treatment discontinuation, and the reason behind it. Patients were classified into three MSKCC and IMDC groups: favorable-, intermediate-, and poor-risk. The required data were collected retrospectively, and the dataset consisted of patients’ demographics, laboratory test results (including complete blood count (CBC), corrected calcium, LDH), treatment delays, treatment duration, and treatment outcomes. The complete blood counts were evaluated before starting the course of treatment. Hematology parameters were measured using Sysmex XN-1000. Laboratory tests were carried out by the Diagnostic Department of the National Research Institute of Oncology. PLR was calculated with the formula [platelet count/lymphocyte count] and NRL with [neutrophil count/lymphocyte count]. Counts of inflammatory cells were taken from laboratory results which were performed immediately prior to the treatment initiation. Detailed information about the laboratory test results at the baseline start can be found in Table 3.

### 2.3. Adverse Events

The initial dosing of cabozantinib was 60 mg per day for all patients. Dose modifications were based on the Summary of Product Characteristics [20]. Adverse events were assessed following the Common Terminology Criteria for Adverse Events (CTCAE) v5.0 [21]. Patients were duly informed of all potential AEs and were actively encouraged to provide details regarding any changes associated with their treatment. Subsequently, AEs were thoroughly assessed at follow-up appointments, which were scheduled every two weeks. Treatment persisted until either disease progression or the onset of significant toxicity, classified as Grade 4 (G4). Any necessary adjustments to the dosage were carefully deliberated in collaboration with the patient, following a comprehensive benefit–risk assessment. A comprehensive record detailing all AEs that manifested during the course of treatment is provided in Table 4.

### 2.4. Statistical Analysis

Categorical variables were summarized with the number and percentage of the respective group. Quantitative variables were summarized with mean and standard deviation (SD; normally distributed) or median, first, and third quartile (Q1; Q3; non-normally distributed), as specified in the Results Section. Progression-free survival (PFS) times were calculated from the date of initiation of cabozantinib (i.e., the start of the study) until the date of diagnosis of progressive disease (PD), death, or were censored on the date of loss to follow-up or the end of the study (5 February 2022). Overall survival (OS) times were calculated from the date of initiation of cabozantinib (i.e., the start of the study) until the date of death or censored on the date of the end of the study (5 February 2022). Survival times were estimated with the Kaplan–Meier method and compared between the groups using log-rank tests. Cox proportional hazard regression was used to verify the associations between patients’ baseline characteristics, laboratory results, adverse events of ponatinib treatment, and survival (PFS and OS). The multiple Cox models were calculated with a backward stepwise method using the predictors significant in a simple analysis. All the statistical tests were two-tailed, and the results were interpreted as significant at *p* < 0.05. Statistica software (version 13; Tibco, Tulsa, OK, USA) was used for computations.

## 3. Results

The study included patients ranging in age between 42 and 80 years, between 4 months and 19 years (237 months) from RCC diagnosis (median 52 months), who were treated between the 30 January 2017 and the 23 June 2021. Thirty (42%) of them were receiving cabozantinib as a second-line treatment (2L), thirty-six (50%) as a third-line treatment, and only five (7%) as a fourth- or fifth-line treatment.

Observation time (from the start of the initiation of treatment with cabozantinib until death or the end of the study on 5 February 2022) was between 1 and 61 months; median (Q1; Q3): 15 months (95%CI: 9; 31). Progression was observed in fifty-five (77%) patients, forty-seven (66%) passed away before the end of the study, and sixteen (23%) patients who did not progress continued cabozantinib at/*to* the end of the study.

The main histological subtype was clear cell RCC (n = 69; 97%). According to International Metastatic Renal Cell Carcinoma Database Consortium (IMDC) criteria, 16 (23%) patients were in the favorable risk group, 45 (63%) were in the intermediate group, and 10 (14%) were in the poor prognosis group. Full characterization of the enrolled patients is shown in Table 1.

Adverse events occurred in almost every patient, with 65 (92%) of them experiencing at least one. The most common AE was hypothyroidism (n = 35; 49%), followed by hand–foot syndrome (n = 33; 46%), hypertension (n = 28; 39%), diarrhea (n = 28; 39%), asthenia (n = 24; 34%), and liver toxicity (n = 11; 15%). Thirty-nine (55%) patients experienced two or more AEs. Dose reduction was necessary in 35 (49%) cases due to toxicity.

**Figure 1:**
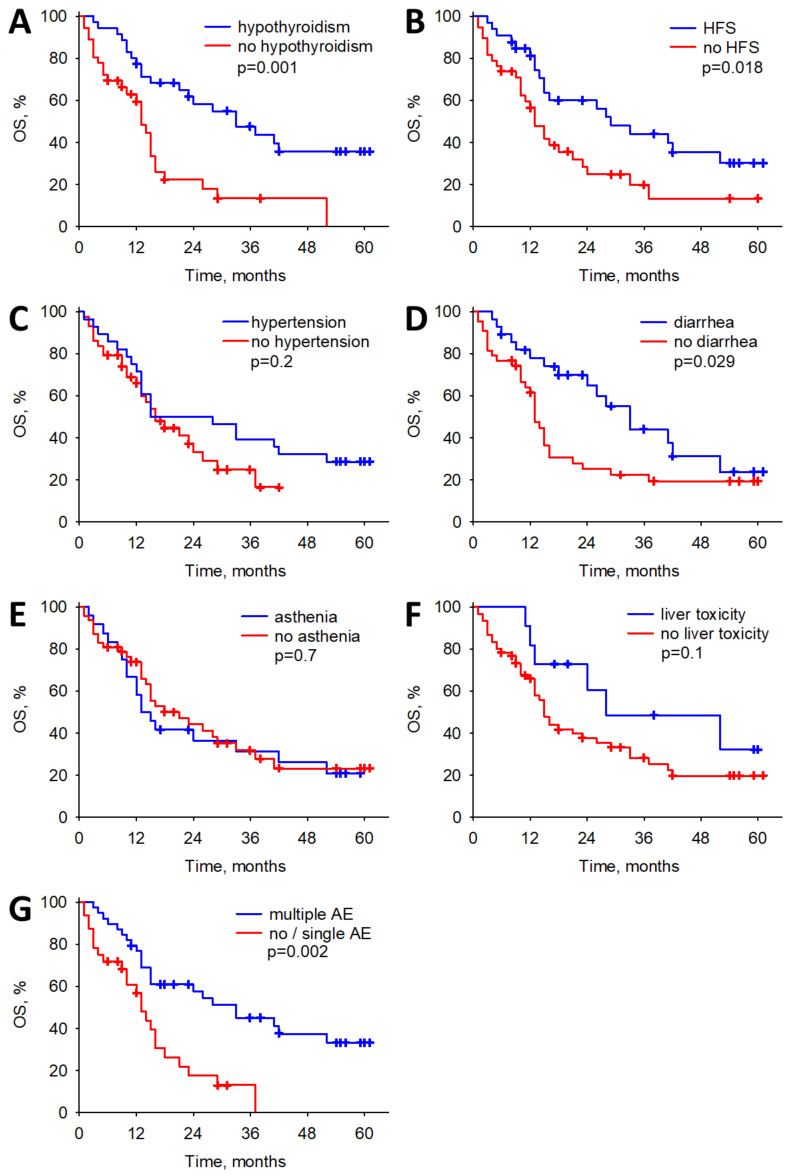
The associations between adverse events (AE) of cabozantinib and overall survival (OS). (**A**) The associations between hypothyroidism and OS. (**B**) The associations between HFS and OS. (**C**) The associations between hypertension and OS. (**D**) The associations between diarrhea and OS. (**E**) The associations between asthenia and OS. (**F**) The associations between liver toxicity and OS. (**G**) The associations between multiple AE and OS. Occurrence of hypothyroidism, HFS, diarrhea and multiple significantly prolongs OS.

**Figure 2:**
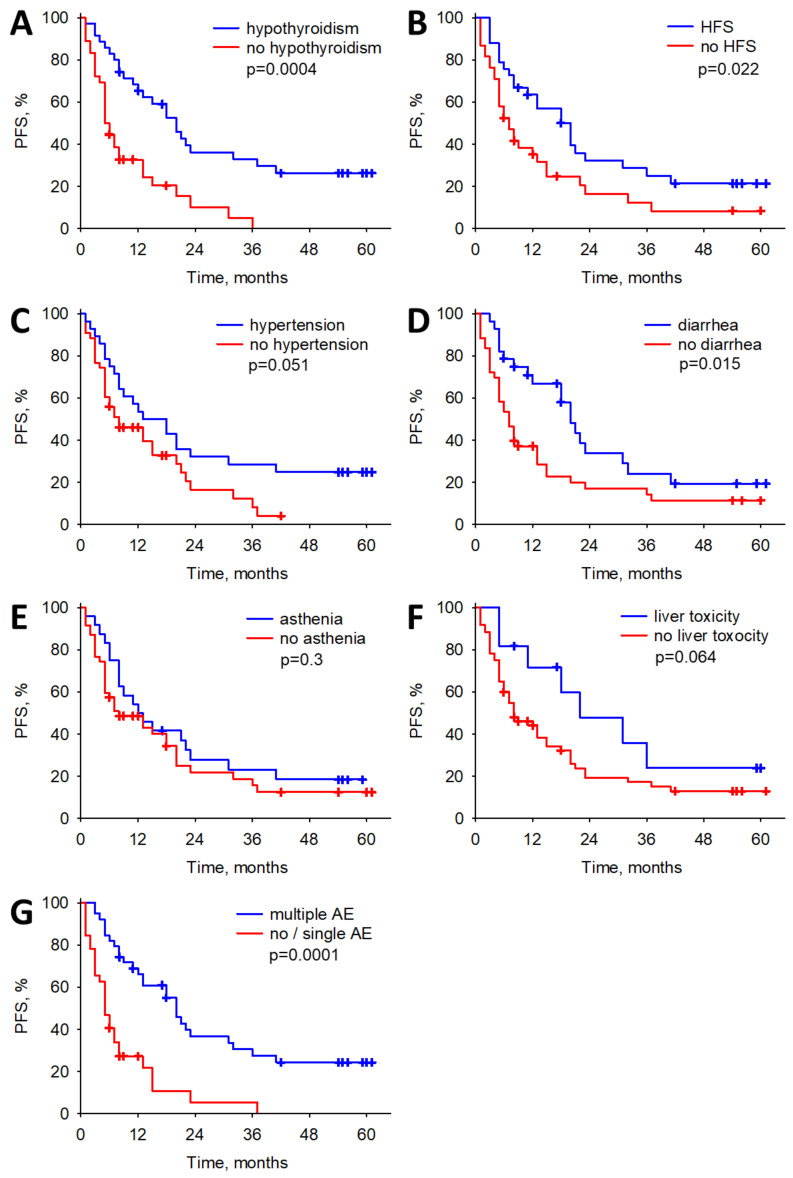
The associations between adverse events of cabozantinib and progression-free survival (PFS). (**A**) The associations between hypothyroidism and PFS. (**B**) The associations between HFS and PFS. (**C**) The associations between hypertension and PFS. (**D**) The associations between diarrhea and PFS. (**E**) The associations between asthenia and PFS. (**F**) The associations between liver toxicity and PFS. (**G**) The associations between multiple AE and PFS. Occurrence of hypothyroidism, HFS, diarrhea and multiple significantly prolongs PFS.

During the course of treatment, the presence of hypothyroidism displayed a statistically significant association with prolonged overall survival (OS) and progression-free survival (PFS) (HR: 0.31; *p* < 0.001, and HR:0.34; *p* < 0.001, respectively), as determined through multiple Cox regression analyses. Likewise, the occurrence of hand–foot syndrome exhibited a noteworthy association with improved OS (HR: 0.46, *p* = 0.021) using the multiple Cox regression analysis model. Furthermore, the presence of diarrhea or hand–foot syndrome (HFS) was correlated with enhanced OS and PFS (HR: 0.53; *p* = 0.039 and HR: 0.49; *p* = 0.02, respectively) using a simple Cox regression analysis model. Patients experiencing multiple adverse events also demonstrated superior OS and PFS compared to those with only one or no adverse events (HR: 0.36; *p* < 0.001 and HR: 0.30; *p* < 0.001, respectively). However, it is noteworthy that hypertension, asthenia, and liver toxicity did not exhibit any significant correlation with improved OS or PFS. All the analyses carried out can be found in Table 5 and Table 6, while a graphical representation of the correlations is shown in Figure 1 and Figure 2.

## 4. Discussion

In this single-center retrospective cohort study, we present real-world data illuminating the prognostic significance of adverse events occurring during cabozantinib treatment among patients with advanced RCC or mRCC who had previously experienced disease progression on the previous line. Our findings indicate that cabozantinib was generally well-tolerated, with no new safety concerns or treatment-related fatalities identified. The comprehensive analysis of adverse events in our study underscores that treatment with cabozantinib consistently leads to the occurrence of at least one such event. The overall incidence of adverse events, irrespective of type and grade, stood at 92%. This observation aligns with findings from the METEOR study, where the incidence rate was 100%, and from the CABOSUN study, where it was 99% [22,23]. More than one AE occurred in 55% of patients. The necessity for dose reduction, observed in 49% of our patients in this study, closely parallels findings from the METEOR and CABOSUN trials, where dose reductions were required in 62% and 46% of patients, respectively. In another real-world experience study conducted by Bodnar et al., the incidence of all AEs was reported at 100% [24], while a study conducted by Iinuma et al. reported an incidence of 79% [25].

An exposure–response (ER) analysis of cabozantinib within the CheckMate 9ER study [26] showed no significant correlation between the extent of exposure to cabozantinib during treatment and progression-free survival (PFS) or risk of death [26]. However, it did unveil a statistically significant association between cabozantinib exposure and the incidence of hand–foot syndrome (HFS) at Grade 1 or higher, as well as severe diarrhea at Grade 3 or higher. Notably, the ER analysis conducted for the METEOR study yielded different results, suggesting a positive correlation between the average concentration of cabozantinib and improved PFS. These differing conclusions can likely be attributed to variations in study designs. Specifically, in the METEOR study, the exposure-response analysis involved simulating cabozantinib concentrations in patients receiving doses lower than 60 mg, whereas the CheckMate 9ER analysis was based on individual average plasma concentrations of the drug. Additionally, it is important to consider that patients in the METEOR study received cabozantinib as monotherapy, while those in the CheckMate 9ER trial were administered a combination of nivolumab and cabozantinib. Furthermore, it is worth noting that in the CheckMate 9ER trial, the median time elapsed before the first-level dose reduction was significantly longer, at 106 days, compared to the METEOR study, where it occurred at 55 days [22].

In other real-world studies with cabozantinib, researchers did not delve into how the occurrence of AEs might affect PFS or OS. However, similar analyses have been conducted with other TKIs, such as sorafenib or sunitinib, which have demonstrated an enhancement in both OS and PFS when AEs like palmar–plantar erythrodysesthesia, hypothyroidism, and hypertension occurred [17,27,28,29,30,31,32].

In this study, approximately 46% of the patients experienced HFS—a common symptom associated with systemic treatment using classical cytostatic agents [33] or targeted tyrosine kinase inhibitors [17,34,35]. The development of HFS is closely tied to the impact of TKIs on endothelial and fibroblastic cells [30]. The inhibition of VEGF-R can impair wound healing, particularly in areas subjected to high pressure and repeated trauma, making them more susceptible to HFS [36,37]. Notably, in this study, HFS was linked to a 50% reduction in the risk of both PFS and overall OS, consistent with findings from similar studies assessing other TKIs [17,38,39].

Numerous theories surround the topic of TKI-induced hypothyroidism; however, the precise mechanism remains a subject of ongoing investigation and is not yet fully understood [40]. It appears to be closely associated with the inhibition of VEGF-R as it is responsible for proper blood flow, which is crucial for the functioning of the thyroid gland. VEGF-R inhibition can also lead to tissue ischemia potentially resulting in thyroid dysfunction [41,42]. In this study, hypothyroidism occurred in 35 patients (55%), making it the most prevalent adverse event. Interestingly, the occurrence of this side effect was associated with a reduction in the risk of PFS and OS by nearly 70%. Some studies have shown statistical significance only for PFS, failing to demonstrate the same significance for OS [43,44,45]. However, several studies have confirmed statistical significance for OS [27,46,47], and Schmidinger et al. established a correlation between hypothyroidism and overall response rate (ORR) [48]. While these aforementioned studies primarily focus on sunitinib and sorafenib, similar associations can also be observed in cabozantinib therapy.

It is important to acknowledge the fact that most patients undergoing treatment with cabozantinib have previously received some form of radical treatment. The resulting reduction in the number of active nephrons makes these patients more susceptible to chronic kidney disease and acute kidney injury (AKI). AKI, characterized by a considerable mortality rate, substantially restricts therapeutic options [49]. Timely management of side effects is crucial, underscoring the importance of the study by Allinovi et al., which describes biomarkers that may be useful in detecting patients at high risk of AKI and in limiting the progression of renal failure [50]. Notably, assignment to a risk group can be made at the stage of radical treatment, and the method is non-invasive. These risk stratification possibilities are particularly valuable due to the potential of cabozantinib to cause rhabdomyolysis, which in turn may contribute to the development of AKI, putting the patient’s life at risk [51]. While a comprehensive examination remains crucial, these biomarkers offer valuable insights, especially in the early stages of emerging renal complications.

Interestingly, in the context of this study, we identified a notable pattern where the presence of multiple AEs emerged as a favorable predictive factor. This intriguing observation presents a dual challenge for both patients and clinicians. Hence, the swift and effective management of AEs becomes imperative, enhancing patients’ ability to endure the therapy successfully [52]. Clearance of cabozantinib varies across the population [26], necessitating dose adjustments for individuals with lower clearance due to their increased susceptibility to developing multiple or severe AEs. Importantly, it has been reported that there is no significant disparity in terms of PFS or OS between patients who underwent a dose reduction to 20 mg and those who maintained the standard 40 mg dose.

Some limitations of this study include the small size of the subgroups, the use of descriptive statistics, and the retrospective nature of the research. Further studies should be conducted to assess the other correlations between adverse events and PFS, OS, or ORR. Uncovering and reporting these correlations can contribute to the development of novel prognostic factors and ultimately provide more informed and personalized patient care. Our findings, therefore, serve as a valuable reference point for future real-world studies focusing on metastatic renal cell carcinoma (mRCC).

## Figures and Tables

**Table 1:** Characteristics of studied group at the start of the study (= start of cabozantinib treatment).

Characteristic	Values Observed in mRCC Patients (*n* = 71)
male sex, *n* (%)	46 (65)
mean age (SD), years	63 (9)
median time from RCC diagnosis (Q1; Q3), years	4.3 (2.0; 8.2)
mean BMI (SD), kg/m^2^	28.1 (5.9)
morphology	
clear cell, *n* (%)	69 (97)
non-clear cell, *n* (%)	6 (8)
sarcomatoid differentiation, *n* (%)	11 (14)
nephrectomy, *n* (%)	69 (97)
Fuhrman grade	
1, *n* (%)	6 (8)
2, *n* (%)	33 (46)
3, *n* (%)	21 (30)
4, *n* (%)	11 (15)
MSKCC score	
0, *n* (%)	19 (27)
1, *n* (%)	36 (51)
2, *n* (%)	15 (21)
3, *n* (%)	1 (1)
IMDC prognostic score	
0, *n* (%)	16 (23)
1, *n* (%)	30 (42)
2, *n* (%)	15 (21)
3, *n* (%)	7 (10)
4, *n* (%)	3 (4)
metastases	
lungs, *n* (%)	53 (75)
bone, *n* (%)	24 (34)
liver, *n* (%)	12 (17)
pancreas, *n* (%)	6 (8)
other sites, *n* (%)	31 (44)
median number of sites (Q1; Q3)	2 (2; 3)
ECOG performance score	
0, *n* (%)	19 (27)
1, *n* (%)	42 (59)
2, *n* (%)	9 (13)
3, *n* (%)	1 (1)
Karnofsky performance scale	
100, *n* (%)	10 (14)
90, *n* (%)	21 (30)
80, *n* (%)	37 (52)
<80, *n* (%)	3 (4)
cabozantinib as 2nd-line treatment, *n* (%)	30 (42)
cabozantinib as 3rd-line treatment, *n* (%)	36 (50)
cabozantinib as 4th- or 5th-line treatment, *n* (%)	5 (7)

**Table 2:** Previous treatment (before the initiation of cabozantinib).

	*n* (%)
1st-line treatment	
TKI (sunitinib, pazopanib, sorafenib), *n* (%)	65 (92)
other (immunotherapy), *n* (%)	6 (8)
2nd-line treatment	
TKI (axitinib, sunitinib, pazopanib, sorafenib), *n* (%)	20 (28)
everolimus, temsirolimus, *n* (%)	18 (25)
nivolumab, *n* (%)	3 (4)
3rd-line treatment	
TKI (sorafenib, pazopanib), *n* (%)	4 (6)
nivolumab, *n* (%)	1 (1)
4th-line treatment (nivolumab), *n* (%)	1 (1)

**Table 3:** Results of laboratory test at the start at baseline.

Laboratory Test	Values Observed in mRCC Patients (*n* = 71)
median hemoglobin (Q1; Q3), g/dL	13.1 (11.0; 14.4)
median neutrophils (Q1; Q3), G/L	3.80 (3.20; 5.30)
median lymphocytes (Q1; Q3), G/L	1.73 (1.26; 2.30)
median platelets (Q1; Q3), G/L	252 (198; 343)
median NLR (Q1; Q3)	2.46 (1.53; 3.63)
median PLR (Q1; Q3)	137 (98; 214)

**Table 4:** Adverse events observed in patients and the need for dose reduction.

Variable	Values Observed in mRCC Patients (*n* = 71)
any adverse event, *n* (%)	65 (92)
hypothyroidism, *n* (%)	35 (49)
hand–foot syndrome, *n* (%)	33 (46)
hypertension, *n* (%)	28 (39)
diarrhea, *n* (%)	28 (39)
asthenia, *n* (%)	24 (34)
liver toxicity, *n* (%)	11 (15)
>1 reported adverse event, *n* (%)	39 (55)
median number of adverse events (Q1, Q3)	2 (1–4)
dose reduction	35 (49)

**Table 5:** Predictors of OS in simple and multiple Cox regression.

Variable	Simple Analysis		Multiple Model	
	HR (95% CI)	*p*	HR (95% CI)	*p*
cabozantinib in 3rd line or further (vs. 2nd)	0.50 (0.28–0.89)	0.019	0.49 (0.24–0.97)	0.041
time from RCC diagnosis, per 1 year	0.91 (0.85–0.99)	0.031	not included	
IMDC (score 3 or 4)	2.99 (1.41–6.35)	0.004	2.23 (1.00–5.02)	0.51
hypothyroidism	0.35 (0.19–0.65)	<0.001	0.31 (0.15–0.62)	0.001
hand–foot syndrome	0.49 (0.27–0.90)	0.020	0.46 (0.24–0.89)	0.021
diarrhea	0.53 (0.29–0.97)	0.039	not included	
number of adverse events, per 1 event	0.71 (0.57–0.88)	0.002	not included	
multiple adverse events	0.36 (0.19–0.66)	0.001	not included	
hemoglobin, per 1 g/dL	0.81 (0.70–0.93)	0.003	not included	
neutrophils, per 1 G/L	1.20 (1.03–1.40)	0.017	not included	
platelets, per 100 G/L	1.45 (1.12–1.87)	0.005	not included	
NLR	1.19 (1.05–1.35)	0.007	1.29 (1.12–1.48)	<0.001
PLR	1.005 (1.003–1.008)	<0.001	not included	

**Table 6:** Predictors of PFS in simple and multiple Cox regression.

Variable	Simple Analysis		Multiple Model	
	HR (95% CI)	*p*	HR (95% CI)	*p*
cabozantinib in 3rd line or further (vs. 2nd)	0.34 (0.20–0.59)	<0.001	0.29 (0.16–0.53)	<0.001
IMDC–poor risk (score 3 or 4)	2.13 (1.03–4.41)	0.042	1.58 (0.73–3.41)	0.2
hypothyroidism	0.35 (0.20–0.62)	<0.001	0.34 (0.18–0.65)	<0.001
hand–foot syndrome	0.54 (0.31–0.93)	0.026	not included	
diarrhea	0.52 (0.30–0.92)	0.024	not included	
number of adverse events, per 1 event	0.68 (0.55–0.83)	<0.001	not included	
multiple adverse events	0.30 (0.17–0.53)	<0.001	not included	
hemoglobin, per 1 g/dL	0.80 (0.70–0.91)	<0.001	0.82 (0.70–0.97)	0.017
platelets, per 100 G/L	1.26 (1.001–1.58)	0.049	not included	
PLR	1.004 (1.002–1.007)	<0.001	1.004 (1.001–1.007)	0.016

## Data Availability

The data presented in this study are available on request from the corresponding author (accurately indicate status).
